# Sustainable Drying and Torrefaction Processes of Miscanthus for Use as a Pelletized Solid Biofuel and Biocarbon-Carrier for Fertilizers

**DOI:** 10.3390/molecules26041014

**Published:** 2021-02-14

**Authors:** Szymon Szufa, Piotr Piersa, Łukasz Adrian, Justyna Czerwińska, Artur Lewandowski, Wiktoria Lewandowska, Jan Sielski, Maria Dzikuć, Marek Wróbel, Marcin Jewiarz, Adrian Knapczyk

**Affiliations:** 1 Faculty of Process and Environmental Engineering, Lodz University of Technology, Wolczanska 213, 90-924 Lodz, Poland; piotr.piersa@p.lodz.pl (P.P.); lukasz.adrian@p.lodz.pl (Ł.A.); justyna.czerwinska@dokt.p.lodz.pl (J.C.); artur.lewandowski@p.lodz.pl (A.L.); 238611@edu.p.lodz.pl (W.L.); 2 Department of Molecular Engineering, Lodz University of Technology, Wolczanska 213, 90-924 Lodz, Poland; jan.sielski@p.lodz.pl; 3 Faculty of Economics and Management, University of Zielona Góra, ul. Licealna 9, 65-246 Zielona Góra, Poland; ma.dzikuc@wez.uz.zgora.pl; 4 Department of Mechanical Engineering and Agrophysics, University of Agriculture in Kraków, Balicka 120, 30-149 Kraków, Poland; marek.wrobel@urk.edu.pl (M.W.); marcin.jewiarz@urk.edu.pl (M.J.); adrian.knapczyk@urk.edu.pl (A.K.)

**Keywords:** torrefaction, drying, miscanthus, biocarbon, fertilizers, kinetics

## Abstract

Miscanthus is resistant to dry, frosty winters in Poland and most European Union countries. Miscanthus gives higher yields compared to native species. Farmers can produce Miscanthus pellets after drying it for their own heating purposes. From the third year, the most efficient plant development begins, resulting in a yield of 25–30 tons of dry matter from an area of 1 hectare. Laboratory scale tests were carried out on the processes of drying, compacting, and torrefaction of this biomass type. The analysis of the drying process was conducted at three temperature levels of the drying agent (60, 100, and 140 °C). Compaction on a hydraulic press was carried out in the pressure range characteristic of a pressure agglomeration (130.8–457.8 MPa) at different moisture contents of the raw material (0.5% and 10%). The main interest in this part was to assess the influence of drying temperature, moisture content, and compaction pressure on the specific densities (DE) and the mechanical durability of the pellets (DU). In the next step, laboratory analyses of the torrefaction process were carried out, initially using the Thermogravimetric Analysis TGA and Differential Scaning Calorimeter DSC techniques (to assess activation energy (EA)), followed by a flow reactor operating at five temperature levels (225, 250, 275, 300, and 525 °C). A SEM analysis of Miscanthus after torrefaction processes at three different temperatures was performed. Both the parameters of biochar (proximate and ultimate analysis) and the quality of the torgas (volatile organic content (VOC)) were analyzed. The results show that both drying temperature and moisture level will affect the quality of the pellets. Analysis of the torrefaction process shows clearly that the optimum process temperature would be around 300–340 °C from a mass loss ratio and economical perspective.

## 1. Introduction

Poland as a member of the European Union is active participator in new European Green Deal which will be a revolution in terms of polish energy sector. New regulations in terms of climate and environment are new challenges in upcoming years. The Polish energy sector is currently facing serious challenges such as high energy demand, an inadequate level of infrastructure development production and transport of fuels and energy, extraction of external energy natural gas, receiving crude oil from an external supplier, and, in the long term, environmental protection, given the current climate issues, of which early reactions are strongly related to potential response to the fuel and energy situation. In between the renewable energy sources, thermodynamic and kinetic analysis aspects of Miscanthus torrefaction are characterized by unflagging interest. This paper shows clearly that Miscanthus is a very interesting material, both in pellet production and in further processing for biochar production, used not only as an energy carrier, but also as a new type of carbon source in fertilizer mixtures where it is shown to be a carrier for organic fertilizers. Torrefied Miscanthus are used as an additive for organic fertilizer and pelletized solid biofuel. The aim of this research was to use kinetics data from TGA analysis of the Miscanthus torrefaction process for designing counter-flow reactor for continuous torrefaction process. The presented technique of Miscanthus torrefaction process is based on the assumption that the authors will find out the optimal torrefaction process conditions for Miscanthus (kinetics, residential time of torrefaction process, and torrefaction temperatures in isothermal conditions) as a feedstock in new design installation for conducting continuous torrefaction processing of different energy crops in a semi-pilot scale (steps: Drying Miscanthus with 40% to 5%, torrefaction of Miscanthus with humidity content 5% to 2%). Of all the renewable energy sources, biomass is a CO_2_ neutral biofuel that is used for big-scale heat and electrical energy production, but it can be also used for distributed energy systems and as other bio-based products. Biomass comes from many sources and occurs in the form of shoots of woody plants [1,2,3,4,5] and herbaceous plants [6,7]. As a result of the research on Miscanthus torrefaction process, the author intends to design and build a small installation with a superheated steam counter-current reactor. Products in the form of torrefied Miscanthus will also be able to serve the farmers who produce them, as biochar in the form of an additive (carrier of the carbon element) to organic fertilizer, suitable for agriculture and greenhouses, improving soil properties and increasing productivity. Pomades, fruit stones, kernel shells, and other residuals are also used, such as waste cooking oil [8,9,10,11,12,13]. Recently, so-called water biomass, such as hyacinths or algae, has also become popular [14,15,16]. The classification of biomass according to source and origin is found in ISO 17225-1:2014 [17]. Extensive research is constantly expanding the spectrum to include new types of biomass [18,19,20]. Biomass has relatively uniform energy parameters due to its composition—lignin, cellulose, and hemicellulose [21,22,23], although it is physically very variable [9,24,25,26,27,28,29]. Therefore, it often requires combustion systems dedicated to a given type and form [30,31,32,33]. Another way to use this diversified raw material effectively is to process it into a standardized fuel form such as pellets or briquettes [10,34,35,36]. However, the production processes of compacted biofuels depend heavily on the raw material’s physical properties already mentioned [28,37]. For the quality of the final product (briquette or pellets) to be at the required level, the raw material must first have adequate moisture content. A range of 6–17% is often stated [38], but mostly it is about 10–12% [20,39,40,41,42,43]. The correct fragmentation of raw material is just as important [34,44,45,46,47,48]. Proper preparation of the raw material facilitates high solid density (DE) and mechanical durability (DU), two quality parameters depending on the properties of the raw material, and properly adjusted parameters of the compaction process [48,49,50,51]. Torrefaction is one such technology which, depending on the process parameters, makes it possible to unify the properties of a diversified raw material [2,28,52,53]. Such a standardized product is characterized, for example, by its better grindability [54,55,56,57], and it can be more widely used, not only as a fuel with similar properties to those of coal. Miscanthus is a plant that is often proposed as a promising species for use in energy and not only as a fuel but also for agricultural applications, such as a carrier for organic fertilizers [6,58,59]. Miscanthus has a yield of at least several tons per ha, it requires low expenditure in cultivation, enjoys a simple harvesting technology and it is easy to store. The annual amount of precipitation should be between 400 and 600 mm. This plant tolerates a temporary water deficit well, which is not without significance in the periods of drought occurring in Poland [6]. One of the bio-based products which can be generated from biomass in a true torrefaction process is biocarbon, with a role as a carrier rich in C for the production of bio-fertilizers. Biomass has the biggest potential from all kind of renewable energy sources in Poland. Increasing the biomass share in conventional coal-power plants is a very effective and fast in implementation way of reducing a carbon dioxide and other pollutant gases emission. To increases the biomass share up to 30% or even 50% thermal, the biomass particles must be milled down to sizes where caloric values can be expected. When comparing coal with biomass, of which both are still the dominant solid fuels for heat and electricity production in Poland, often reveal inferior properties of biomass. When we look closely at wooden biomass fuel properties we see that biomass has, in most cases, a high moisture content, resulting in storage complications such as self-heating and biological degradation, lower energy densities, is a bulkier fuel (with poorer transportation and handling characteristics), and more tenacious (the fibrous nature of biomass makes it difficult to reduce to small homogeneous particles). Biomass properties mentioned above have negative impacts during energy thermal conversion such as gasification system design limitations and lower combustion and co-firing efficiencies. As a torrefaction product we get a hydrophobic solid fuel with greatly increased grindability and energy density (on a mass basis). What is more important is that we lower the required energy to process the torrefied biomass and determine that it no longer requires additional separate handling facilities, like when we co-combusted new fuel with coal in operating power plants. It is suggested that torrified biomass can be compacted into high grade pellets with substantial superior fuel properties compared with standard wood pellets from untreated biomass. The carbonization process can be combined together with drying and palletization process, with both energy and economy benefits [60]. In this paper, the torrefaction process of the most promising of energy crops plus low-cost effective feedstock was performed using an electrical furnace in the presence of CO_2_ gas and a thermogravimetric analyzer with two other inert gases, argon and nitrogen. In addition, Miscanthus, with its sorptive and fertile characteristics of torrefied biocarbons, was evaluated with respect to its potential as a carbon sequesterer and soil fertility enhancer [61,62,63,64,65]. Biocarbon as a carrier for natural biofertilizer has a huge potential in farming [66,67] as an additive for bio-fertilizers for greenhouse cultivation, agriculture, and horticulture. One of the main objectives of this research was to establish proper Miscanthus torrefaction process conditions (torrefaction temperature, heating ratio and resident time, kinetics, and energy activation) to design installation with capacity 50 kg/h of wet biomass in a semi-pilot scale with counter-flow torrefaction reactor for continues torrefaction process to produce: Carbonized solid biofuel and biochar for fertilizers. Demand for biocarbon, not only as a bio-product but primarily for the products manufactured in its use, will stimulate an increased interest in biocarbon produced by thermo-chemical conversion in the Polish market: Fuels for biogas plants, sugar production, bioethanol production plant, saw mills, and pulp and paper factories [68,69].

## 2. Results

### 2.1. Analysis of Miscanthus Drying Process and Pellets Properties of Miscanthus

**Figure 1:**
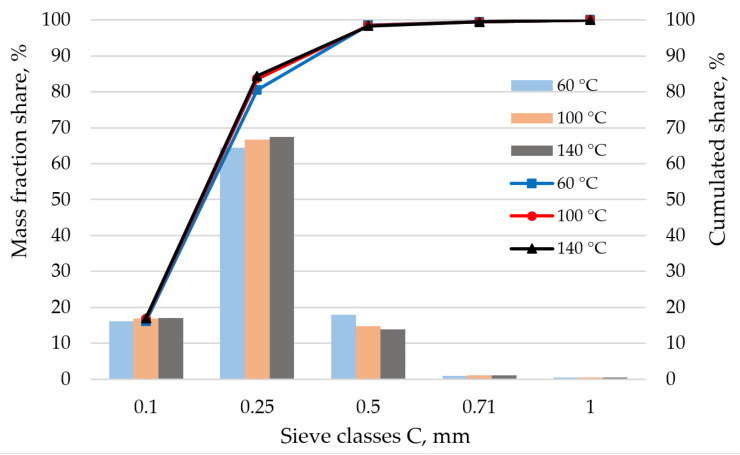
Analysis of samples’ particle size distribution.

Figure 1 shows the particle composition analysis of the samples dried in the temperature range examined. Both the bar graphs, showing the mass shares of sieve class as well as the cumulative graph, indicate that there are no differences in particle size distribution (PSD) between the samples. This shows that the calculated value of d50 for all samples is 0.2 mm. These results help us to conclude that the particle composition did not influence the DE and DU quality parameters of the pellets. The degree of sample fragmentation is at the level recommended by Mani et al. [48].

**Figure 2:**
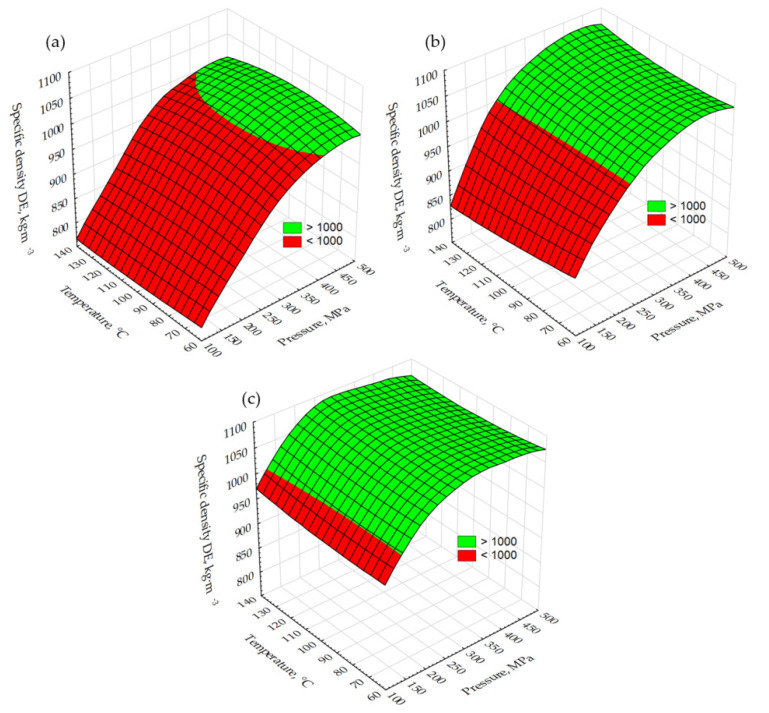
Changes of test pellet specific density DE due to the temperature of drying and compaction pressure: (**a**) dry sample, (**b**) moisture content at a level of 5%, and (**c**) moisture content at a level of 10%.

**Figure 3:**
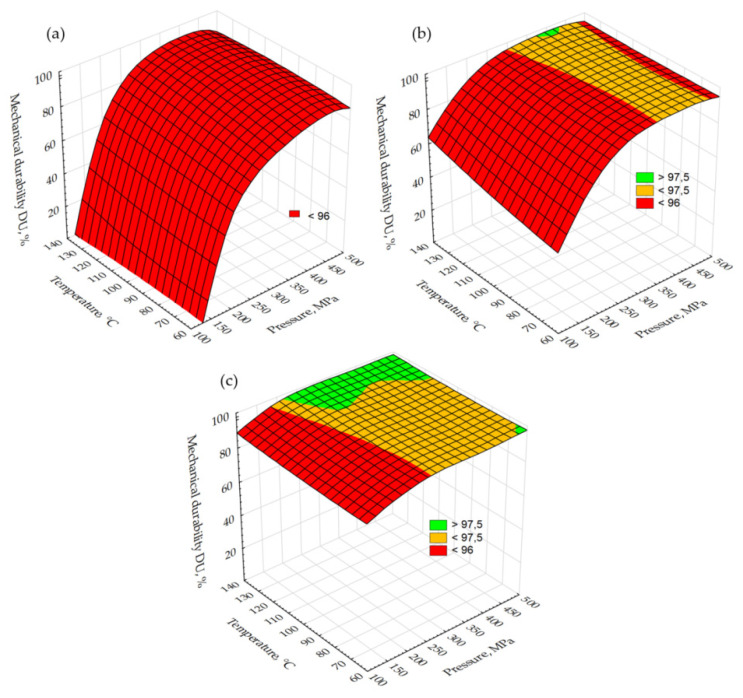
Changes of test pellet mechanical durability DU due to the temperature of drying and compaction pressure: (**a**) dry sample, (**b**) moisture content at a level of 5%, and (**c**) moisture content at a level of 10%.

Figure 2 shows the pellets’ specific density DE changes due to temperature drying and compaction pressure. For the samples of dry material, the DE values increased from 811 to 1008 kg·m^−3^ (Figure 2a). In this case, the lowest pressure at which the threshold was reached (1000 kg·m^−3^) was determined for a sample dried at 100 °C and was about 350 MPa. Samples dried at 60 and 140 °C reached the assumed DE value at about 390 MPa. For samples with 5% moisture content, the DE surface was shifted up and reaches values in a range of 890–1075 kg·m^−3^ (Figure 2b). The DE threshold was reached at 230 MPa for all samples, suggesting that the drying temperature, in this case, was not important, as opposed to the moisture content, the growth of which results in an increase in DE. When it increased to 10%, the DE surface again shifts up (DE range is 990–1090 kg·m^−3^) with a threshold at a pressure level of 140 MPa regardless of drying temperature (Figure 2c). Figure 3 shows pellet changes in DU. Threshold values were set at 96 and 97.5% (quality class B and A for non-woody pellets according to ISO 17225-6:2014 standard). DU thresholds for pellets made from material in a dry state (Figure 3a) were not reached and no effect on the drying temperature could be observed. When the moisture content of the material rose to 5%, the first DU threshold (96%) was reached at pressures of 300 MPa (material dried at 140 °C) and of about 340 MPa (material dried at 100 °C and 60 °C). The second threshold was not reached in this variant except for a small peak at a pressure of 400 MPa for material dried at 140 °C (Figure 3b). A further increase in the moisture content to 10% was significant for an increased pellet DU. The first threshold was reached at 180 MPa (dried at 140 °C) and above 240 MPa for the rest of the samples (Figure 3c). The second threshold was reached only for material dried at 140 °C and at about 230 MPa. The results presented indicate that water (moisture content 5% and 10%) is a binder and leads to improved connections between particles [39,40]. This results in an increase in DE and DU values. Slightly higher DE values for material dried at 140 °C can be caused by the sugars contained in the raw material cells and released due to this temperature. These sugars in combination with moisture act as a binder. However, this needs to be confirmed in further tests.

The statistical analyses carried out allowed the identification of groups between which there are significant statistical differences. Results for DE are presented in Table 1, and for DU in Table 2, with significance level α = 0.05.

**Figure 4:**
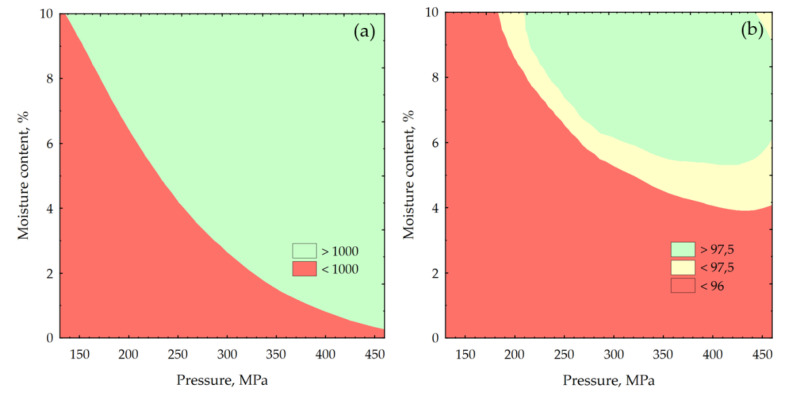
Changes of test pellet quality due to moisture content and compaction pressure: (**a**) specific density and (**b**) mechanical durability.

It was shown that the drying temperature has no influence on DE and has a slight effect on DU. Therefore, a layered graph was made to show how the changes in DE and DU progress in accordance with the moisture content and compaction under pressure (Figure 4).

### 2.2. Kinetic Analysis of Miscanthus Torrefaction Process

**Figure 5:**
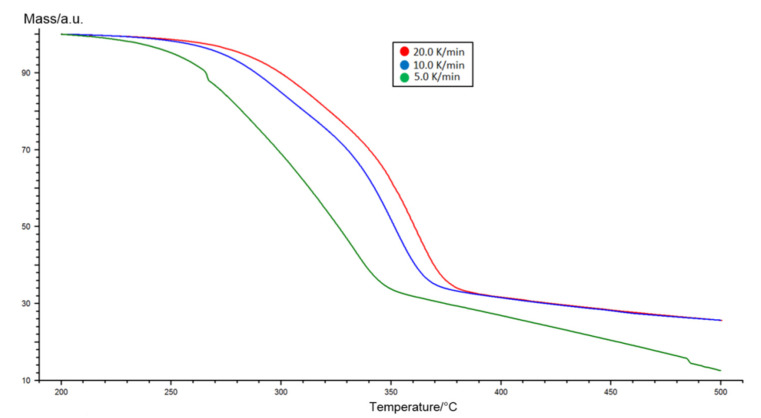
Thermogravimetric analysis results for heating speeds of 5, 10, and 20 K/min determined for Miscanthus.

Thermogravimetric analysis of the Miscanthus torrefaction process was performed using three different heating ratios 5, 10, and 20 K/min. The results of the TGA analysis for Miscanthus are shown in Figure 5.

**Figure 6:**
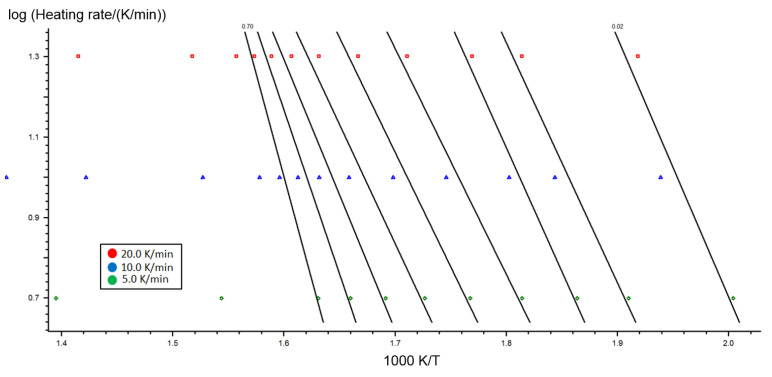
Presentation of the Ozawa–Flynn–Wall logarithm dx/dt function for Miscanthus.

**Figure 7:**
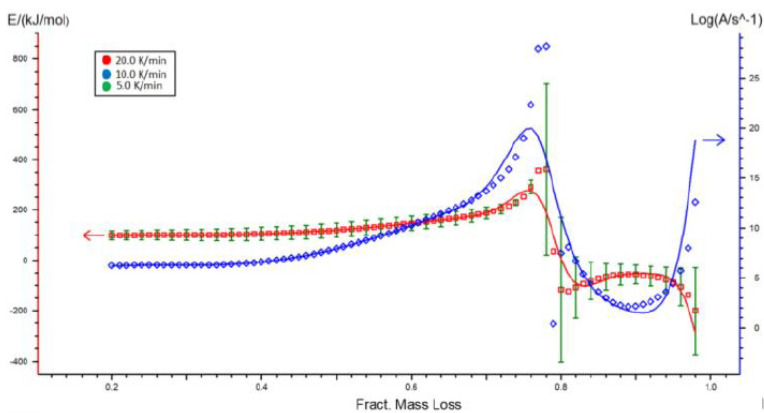
Pre-exponential coefficient and activation energy of Ozawa–Flynn–Walla conversion for Miscanthus.

Making assumptions for the stability f(x) at a given level of conversion, this technique enables the logarithm dx/dt to be plotted as a function of temperature inverse, and the activation energy (Figure 6) and pre-exponential coefficients to be estimated for each level of conversion while not knowing the reaction mechanism (Figure 7). Table 3 contains the activation energy and pre-exponential coefficient as a function of the level of conversion by the Ozawa–Flynn–Walla method.

**Figure 8:**
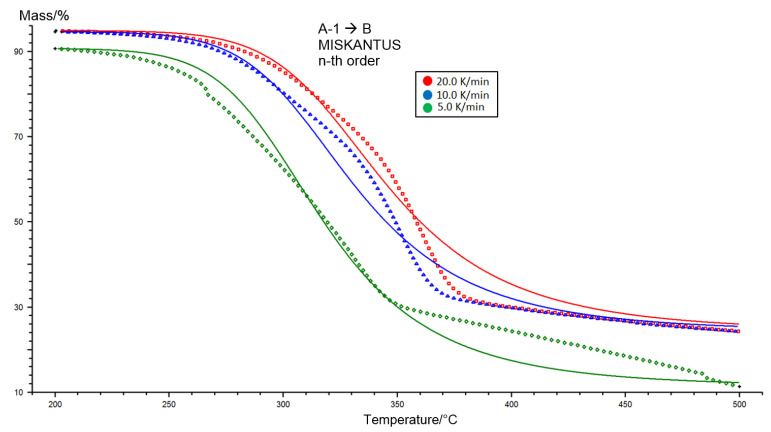
Fitting the calculated kinetic model to the experimental data for the Miscanthus.

Using the Netzsch Kinetics 3 program, based on thermogravimetric TG curves estimated as a function of temperature, a mathematical adjustment of one thermal depolymerization reaction model was made. By using the estimated reaction model, kinetic parameters were delivered, that is to say, the velocity constant (pre-exponential factor) k and activation energy E_a_, in addition to the fitting factor R^2^ in Table 4. The most optimal fit for the experimental data for the Miscanthus sample was found for the n-order reaction model shown in Appendix A, Table A1 for *n* = 2.9. The correlation coefficient R^2^ was 0.9932, the pre-exponential coefficient 10.42, and the activation energy 142.52 kJ/mol. Figure 8 represents the fit of the equation with estimated coefficients fitted to the experimental data.

One important fact that has to be remembered is that the estimated kinetic trio is only the optimal mathematical fit of the equation to the experimental data, and in this case there is no exact physical significance (reaction order). By using the above fitting procedure, the activation energy, thus delivered, is visible: It is only used to correlate the model with the experimental data and does not matter with reference to the definition. The conclusions are that kinetic analysis methods employing the results of different heating ratios were more suitable than a single heating ratio method.

## 3. Discussion

### 3.1. TGA Analysis

**Figure 9:**
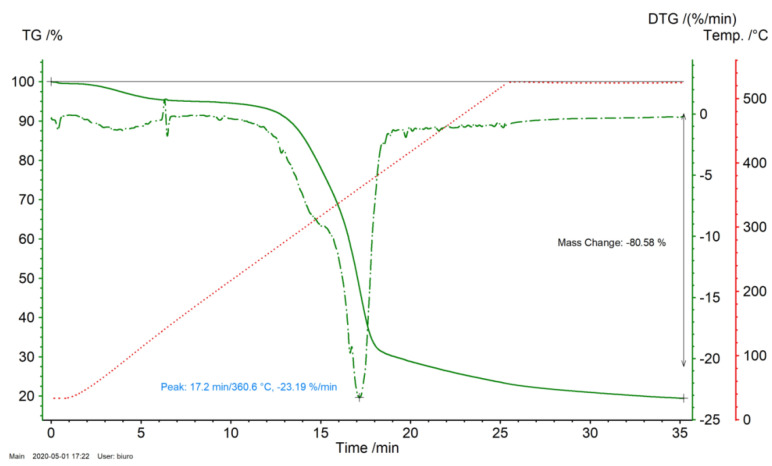
Thermogravimetric analysis of the Miscanthus torrefaction process for the production of biocarbon at a temperature of 525 °C (heating ratio 20 K/min) in an N_2_ atmosphere.

A TGA analysis of Miscanthus was performed in an inert atmosphere N_2_ using TGA Netzsch Tarsus 209 FC (Netzsch, SelbBavaria, Germany). In Figure 9 we see the derivative thermogravimetric Differential Thermogravimetric DTG and thermogravimetric TG analysis results of the Miscanthus sample torrefied in an N_2_ atmosphere.

The first phase of the Miscanthus roasting process can be detected at temperatures of approximately 257 °C because of the thermal depolymerization and destruction of the organic compounds which contain the Miscanthus. The thermal depolymerization of the Miscanthus was stopped at 525 °C. A mass reduction resulted in a lower energy yield. In this research, the focus was on the determination of apparent kinetic parameters, and it is of great use for the engineering design stage of the installation of both a biomass regimental dryer and a reactor. The main focus of this work was not on conducting research to get to know the fundamental chemical mechanisms of the torrefaction of Miscanthus.

### 3.2. DSC Analysis

**Figure 10:**
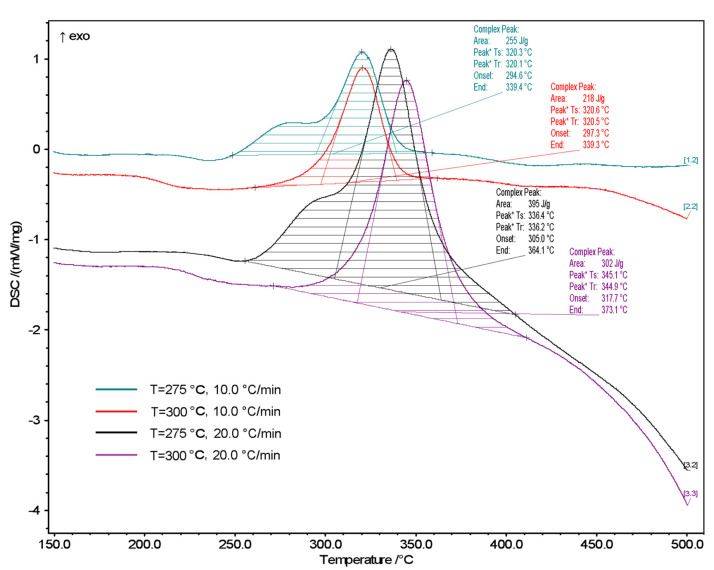
DSC analysis of torrefied Miscanthus 257 °C and 300 °C (heating ratios: 10, 20 K/min) in an N_2_ atmosphere.

The torrefaction process initiation temperatures along with the heat liberated at different heating ratios were calculated from the DSC curves. The initiation temperature rises together with increasing heating ratios, and this is in agreement with the data presented in Figure 10. The initiation temperature increased by nearly 20 °C when increasing the heating ratio from 10 to 20 °C/min. The heat liberated during the torrefied Miscanthus (torrefaction temperature; 257 °C) combustion increased by 995 W/g when increasing the heating ratio from 10 to 20 K/min. In Figure 10 we can see the DSC pyrolysis curves of torrefied Miscanthus at different heating ratios of 10, and 20 K/min in an N_2_ atmosphere in order to investigate the pyrolysis of torrefied Miscanthus in different temperatures. Decomposition of the first component (hemicellulose) of the torrefied Miscanthus, followed by a broad exothermic pyrolysis peak of the mixture of three principal constituents that shifted to a higher temperature with an increasing heating ratio from 10 to 20 K/min. The torrefied Miscanthus pyrolysis decomposition peaks appeared at 320.3, 320.6, 336.4, and 345.1 °C with heating ratios of 10 and 20 K/min, respectively (torrefaction temperature; 257 °C). Le Brech et al. [48,50] have studied the combustion of nearly 30 common biomass types, including Miscanthus, and they reported that Miscanthus showed two temperature ranges of combustion of 240–340 and 450−550 °C using the DTG data. Furthermore, they found that the combustion characteristics of the lignocellulosic biomass are about the same, and thus they proposed that the two steps of the kinetic reaction are:

X(solid)→Y(solid) + G1(gas) Step 1

Y(solid)→A(ash) + G2(gas) Step 2

### 3.3. C, H, N, O, S Analysis

In Table 4 the results of a proximate analysis of Miscanthus before and after the thermochemical conversion process are presented. It is quite clear that the C% (weight) in the biomass thermochemical process bio-products increases in tandem with an enhanced Miscanthus torrefaction process temperature. This is contrary to the weight percentages of H and O which showed a decreasing trend. We can conclude from the above mechanism that dehydration takes place as well as decarbonization during the Miscanthus torrefaction process. This clearly shows that a decrease in the H and O contents of torrefied biomass will result from the emission of CO_2_, CO, or H_2_O [22]. A rising percentage of the C content is due solely to a decrease in the O content [25,26,36,37].

### 3.4. Energy Yield, Mass Yield, Volatile Matter, Ash Content, High Heating Value

In Figure 10, TGA Miscanthus carbonization process curves are shown with green points in the drawing. The Miscanthus torrefaction process was performed up to 525 °C for 9 min where the samples were kept under isothermal conditions. As is evident, the percentage of Miscanthus mass reduction was 78.80%. The mass decreased by 0.13% while the samples were held under isothermal conditions at 525 °C. As seen in Figure 10, it was at about a temperature of 80 °C where the initial evaporation of moisture from the sample occurs. In the case of carbonization up to 257 °C, the maximum heating ratio was 2.1%/min at 266 °C (the maximum instantaneous temperature of this process during stabilization to 257.5 °C). The maximum carbonization process speed for Miscanthus up to 300 °C was 5.16%/min at 300 °C, and in the case of thermochemical conversion up to 525 °C, the maximum process speed was 10.42%/min at a temperature of about 337 °C.

**Figure 11:**
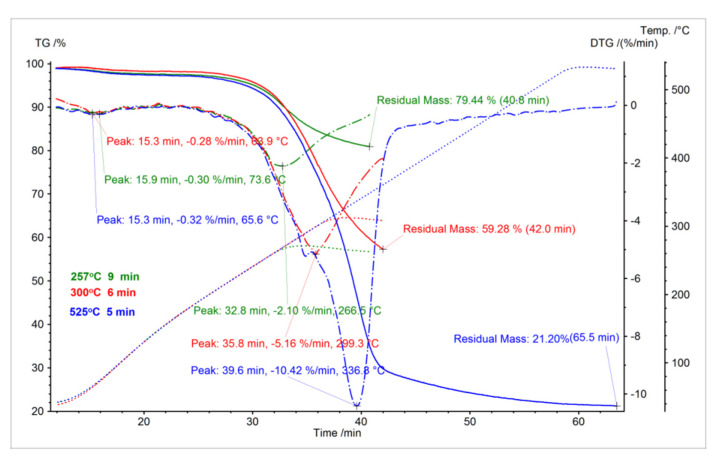
Analyses of Miscanthus mass loss with DTG curves during the torrefaction process.

Figure 11 shows the trajectory of torrefaction curves. In the drawing, the blue points show all carbonization process curves representing the end of the heating process and the first stage of maintaining the roasting sample at a specific temperature. Miscanthus thermochemical conversion was conducted up to 257 °C and with a residential time under isothermal conditions equal to 9 min, and it was evident that the Miscanthus mass reduction stood at 10.62%. However, the mass reduction was 11.68% after a period of 6 min at 300 °C, and when it was held under isothermal conditions at 525 °C the mass reduced by 0.13%. A set of maximum process speeds (minima on DTG curves) were given alongside a set of TG-DTG. As is evident in Figure 11, the initial evaporation of moisture from the Miscanthus straw sample occurs at the quickest speed at around 70 °C where torrefaction takes place up to 257 °C. The maximum Miscanthus torrefaction process speed was 8.01%/min where the temperature was around 300 °C and for torrefaction up to 525 °C the maximum process speed was 8.81%/min where the temperature was 305 °C.

As can be seen, the Miscanthus weight decrease was 2.10%/min when the holding time under isothermal conditions was 6 min at 300 °C in the Miscanthus torrefaction process carried out up to 257 °C while keeping the sample 9 min under isothermal conditions. In addition, the weight decrease was 5.16%/min while when holding the sample at 525 °C, and the mass reduction was 10.42%/min. 

The torrefaction process was performed on Miscanthus to establish the best torrefaction temperature for a variety of industrial applications such as carbonized solid biofuel, a carrier for bio-fertilizers, and activated carbon. Pyrolysis is the term used where the thermal treatment is above 300 °C. Visible rises in the high heating value (HHV) up to 400 °C are connected to the removal of oxygen and hydrogen. There is a reduction in the HHV when the temperature rises above 500 °C. In Table 5 there is presented content of mineral elements in the torrefied Miscanthus and untreated Miscanthus at three different temperatures.

The torrefied Miscanthus produced as a result of ecological fertilization is characterized by low cultivation costs and the heat energy produced in this carbonized solid biofuel requires about 25% less expenditure than during fertilization with chemical fertilizers.

**Figure 12:**
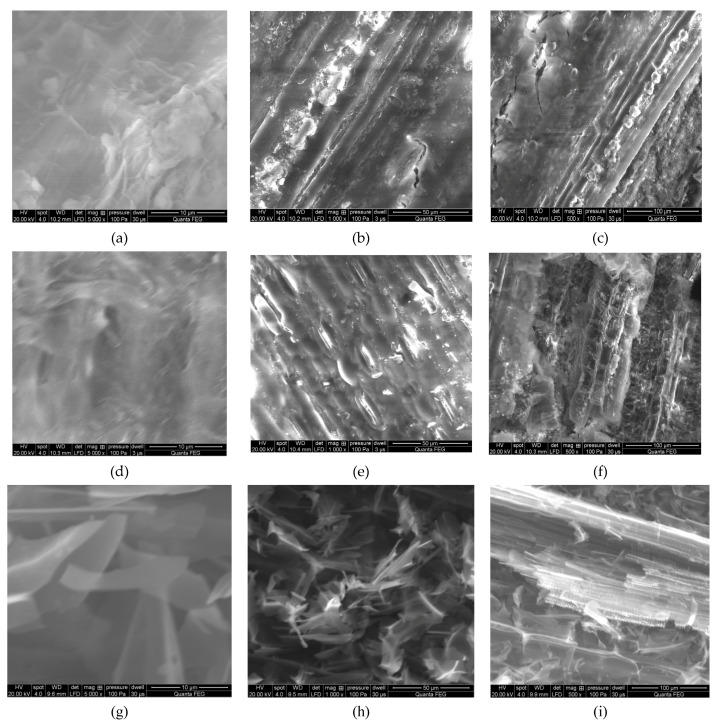
SEM microscopic images of torrefied Miscanthus: 257 °C magnification (**a**) 5000×, (**b**) 1000×, and (**c**) 500× and 300 °C magnification (**d**) 5000×, (e) 1000×, (**f**) 500×, 525 °C (**g**) 5000×, (**h**) 1000×, and (**i**) 500×.

Figure 12 SEM microscopic images of torrefied Miscanthus in three different temperatures. We can see how the –OH groups are degraded with torrefaction process temperature increase. This article presents results showing that the energy yield drops slowly from 90% to 33% as the temperature of the Miscanthus carbonization process increases. During the experiment, reducing the weight of Miscanthus during the roasting process at 300 °C shows the nearest weight reduction to 50%. Miscanthus was then held for 6 min, and the weight reduction corresponding to this residence time was 40.72%. These data are used to calculate the profitability of using Miscanthus as a carrier for bio-fertilizers. A high carbonization rate and low energy input in combination have the most significant impact on the economics of the torrefaction process. Therefore, it was assumed that the use of Miscanthus as a carrier for bio-fertilizers after roasting should not exceed 50% in weight reduction. So that this can be achieved, the holding time needs to be as low as possible. In order to produce activated carbon, the weight loss should not exceed 75%, while during thermochemical conversion of Miscanthus at 525 °C it was noticed that the residence time with the shortest period was 5 min, and the weight loss was 78.80%.

**Figure 13:**
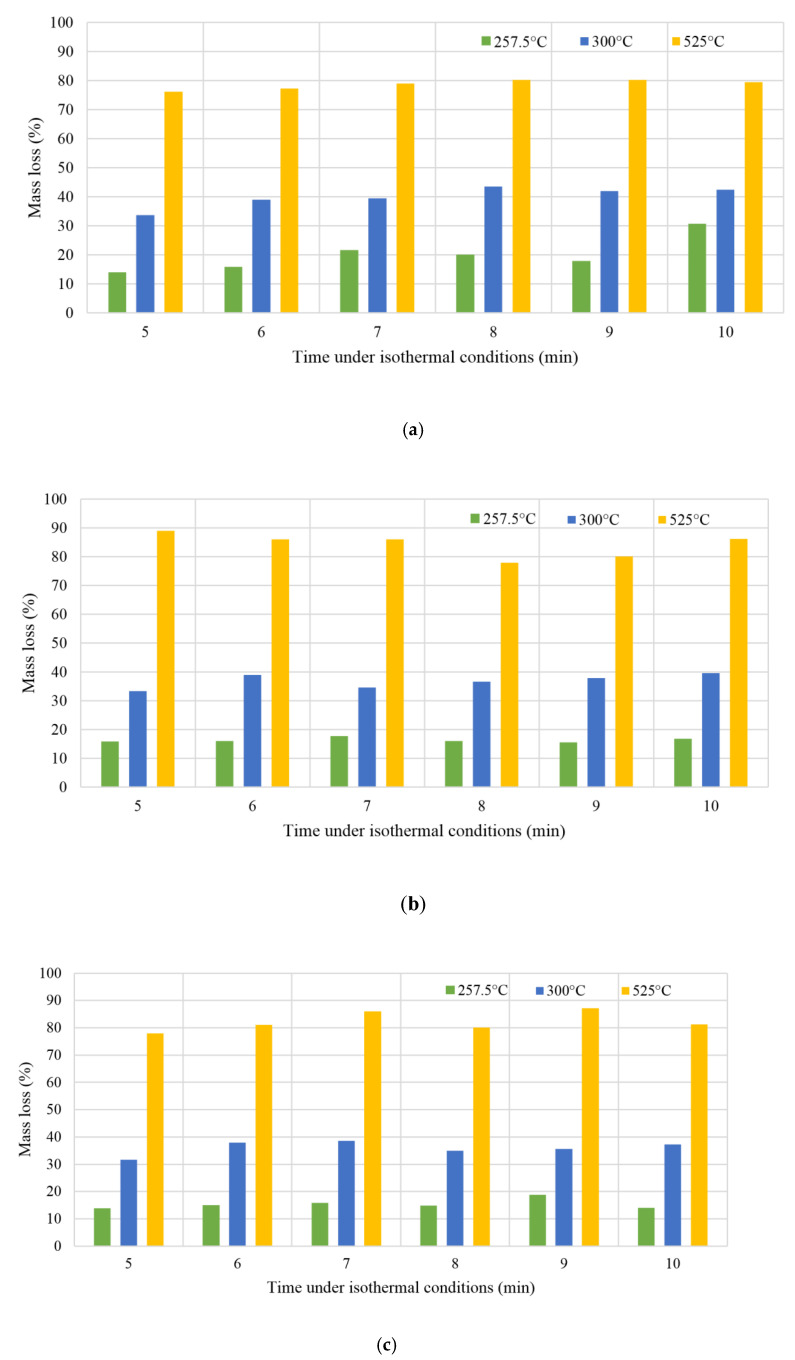
Torrefaction process of Miscanthus: Thermogravimetric analysis results and optimal process conditions for the production of carbonized solid biofuel, carrier for natural fertilizer and activated carbon (Figure 13 heating ratios: (**a**) 5 K/min, (**b**) 10 K/min, and (**c**) 20 K/min).

Figure 13 shows that the mass residua of Miscanthus torrefaction products proceed at three temperatures, 257.5 °C, 300 °C, and 525 °C, in residential times from 5 to 10 min (under isothermal conditions) using three different heating ratios, 5, 10, and 20 K/min. Three heating ratios were deployed, and data were checked on the elemental composition and thermal stability of the isotherm research material. It shows a different mass loss ratio in correlation with different residential times. More detail analysis of the results presented at Figure 13 was described in Section 3.5 Biomass Torrefaction Residence Time and its effect on the process.

At 300 °C, an alteration may be observed, which indicates a substantial variance between the values before and after the biomass carbonization process, a maximum mass loss of 44.10%, and minimum mass loss of 32.38%. In spite of that, the residual masses at 257.5 °C are the highest of all recorded in the thermogravimetrical analysis. This is an excellent result that demonstrates mass reduction with an increase of temperature: The mass percent of the biomass at 525 °C is the lowest of all measurements. When the isotherm is extended at 300 °C, the Miscanthus mass decreases. Dynamic and isothermal measurements at a 10 K/min heating ratio were made on samples of Miscanthus. This yielded data on the thermal stability and the elemental composition of the isotherm research material. This was divided into five samples, from 5 to 10 min, according to the measurements found at the three temperatures of 525, 300, and 257 °C [63]. As a result of these studies, it was found that the most optimal torrefaction temperatures for Miscanthus for the production of a biocarbon as a carrier for fertilizers lie between 300 and 340 °C looking at it from a mass loss ratio and economical perspective. However, the temperature may differ in practice based on the initial state of the raw material, the heating ratio, and the chemical composition of the raw biomass [2]. Researchers from Norway who have been working on biochar use as an additive for bio fertilizers have found that persistent biochar C with a half-life 60 times higher than the parent feedstock can be achieved at pyrolysis temperatures as low as 370 °C, with no further gains to be made at higher temperatures. Biochar produced from Miscanthus and corn cob re-applied to soil previously incubated at the highest temperatures was mineralized faster than when applied to non-incubated soil. Their results from a 1-year incubation study show that biochars with long residence time in soils can be produced from Miscanthus and corncob feedstocks when a threshold temperature requirement of 370 °C is reached, and there is no gain in resistance beyond this value. It is also important to mention that they state that the mechanisms behind these effects need to be better understood if we want to predict the long-term stability of biochar in soil.

### 3.5. Biomass Torrefaction Residence Time and its Effect on the Process

A thermogravimetric analysis of the Miscanthus torrefaction process contributed to the observation that biomass thermochemical conversion temperatures above 300 °C resulted in decreasing energy and mass yield. In Figure 13a correlation can be seen between the effects of the Miscanthus torrefaction residual time and the characteristics of the Miscanthus carbonization process. It was observed that the result of thermochemical conversion residual time on volatile compounds in terms of ash content was not as large as that of the Miscanthus carbonization process conditions such as temperature. In Figure 13, we observed that the mass yield reduces when holding time increases. The decrease in the water content and volatile matter of the Miscanthus provides an explanation for this. In spite of that, there was a very high mass loss at the start of the biomass torrefaction process while the change of mass yield was not so meaningful with regard to a longer torrefaction residence time. The explanation for this lies in the degradation of more reactive biomass elements at the first stage of the torrefaction process. From Figure 13 it is possible to infer that the HHV increases with Miscanthus torrefaction time. Nevertheless, in research performed thus far, the Miscanthus thermochemical conversion temperature is more significant than residual mass.

### 3.6. Volatile Organic Content (VOC) Analysis

When Miscanthus carbonization was conducted at temperatures up to 300 °C it was found that mass loss approximated to that of the untreated Miscanthus before the thermochemical conversion process indicated by the mass yield of Miscanthus. This clearly demonstrated that the extent of torrefaction for Miscanthus up to 300 °C was small when equated to values above 300 °C.

**Figure 14:**
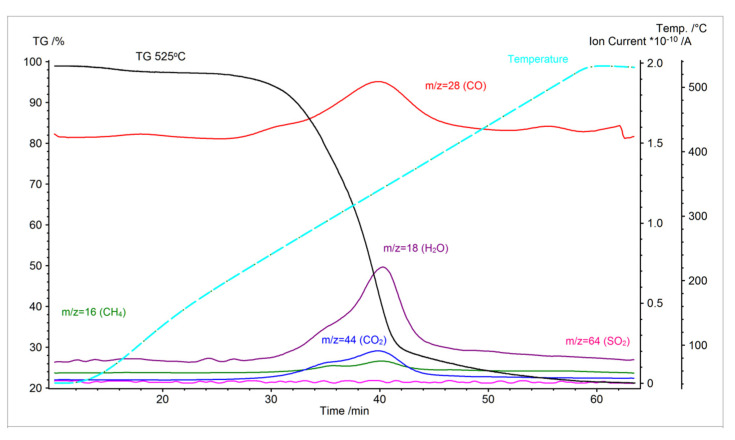
Thermogravimetric curve and ion current curves for Miscanthus thermochemical conversion performed at 525 °C.

The first visible weight loss is closely related to the moisture loss in the first stage of the process (the fastest at 64–74 °C to 110 °C), and the second loss is thermal decomposition, which creates volatile organic compounds such as water vapor, carbon monoxide, carbon dioxide, acetic acid, and other organic substances (Figure 14). This effect can be observed in moisture loss and then in the depolymerization of secondary cell wall components, hemicellulose, cellulose, and lignin, as can be seen in Figure 13. Weight reduction during thermochemical conversion at lower temperatures can mainly be explained by H_2_O loss. In the process of torrefaction of Miscanthus at a temperature over 300 °C, the reduction is associated with the thermal decomposition of this energy crop. The key effect that was observed during this process is the increase in volatile matter content, while ash content was reduced where temperatures were over 525 °C. The HHV increases constantly with a rise of volatile compounds and the decrease of ash content.

**Figure 15:**
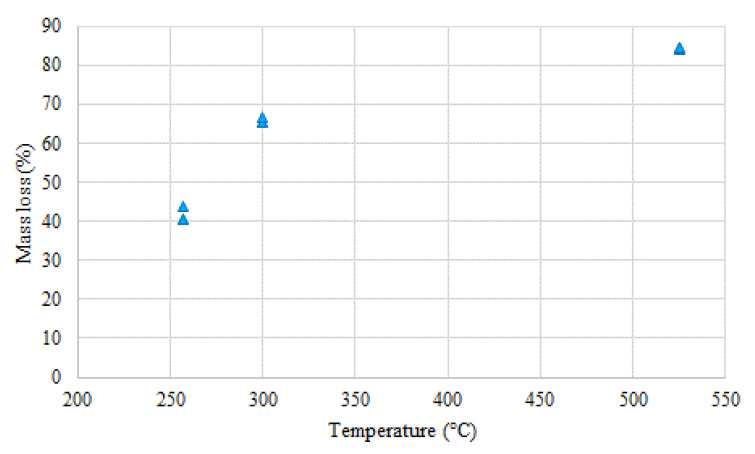
Mass loss of Miscanthus after a torrefaction process in a CO_2_ atmosphere using an electrical furnace.

**Figure 16:**
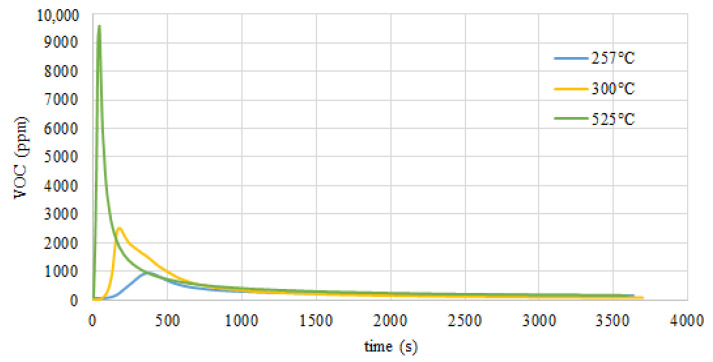
Volatile organic content (VOC) emission in ppm of the Miscanthus torrefaction process in an electric oven in a CO_2_ atmosphere.

Figure 14, Figure 15 and Figure 16 show the results of volatile organic measurements for Miscanthus. The research results found that a Miscanthus carbonization process carried out below 300 °C is more suited to producing solid biofuels. Hydrocarbon emissions were observed above 370 °C for the torrefaction process, with a direct and negative impact on torrefaction energy efficiency. As can be seen in Figure 11, between 260 and 280 °C the most active thermal-chemical conversion temperatures for Miscanthus are disturbed in order to obtain a size difference of 45–50%. At temperatures exceeding 300 °C, HHV remained at 24 MJ/kg in this range, and the increase in energy was maintained above 70%. Energy efficiency in relation to HHV remains at the most optimal level at a higher range. The area of carbonization changes in cases of new physico-chemical properties of the primary material and heating speed [64].

**Figure 17:**
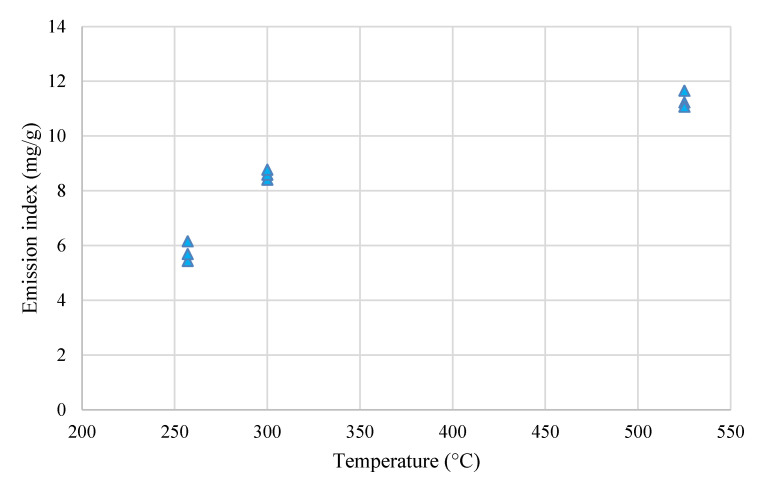
Emission index for the Miscanthus torrefaction process using an electric oven.

On Figure 17 emission index for the torrefied Miscanthus in electric oven is presented which is defined as: The amount of pollutants emitted in relation to the amount of fuel subjected to a specific process. The emission factor is given in mg of a given pollutant referred to one gram.

## 4. Materials and Methods

### 4.1. Drying and Pelletizing Methods

This research concentrated on the drying and torrefaction process of one energy crop, Miscanthus. Stems were harvested in the winter of 2019/20, chipped, and then sieved. The study was conducted on chips that went through a 16 mm sieve and remained on an 8 mm sieve. Separation was carried out on a shaker (LPzE-4e, Morek Multiserw, Marcyporęba, Poland). Specimens were then split into three samples and then dried (SLW 115 dryer, Pol-Eko, Wodzisław Śląski, Poland) at temperatures of 60, 100, and 140 °C until they reached a dry state. After that, all of them were milled using a hammer mill with a 1 mm hole size bottom screen (PX-MFC 90D Polymix, Kinematika, Luzern, The Switzerland). For each sample, PSD was determined in line with the ISO 17827-2 standard [70]. Samples were divided into 5 sieve classes, C_1_: 0.1—diameter of particle d ≤ 0.1 mm, C_2_: 0.25—diameter of particle in the span 0.1 < d ≤ 0.25 mm, C_3_: 0.5–0.25 < d ≤ 0.5 mm, C_4_: 0.71–0.5 < d ≤ 0.71 mm, and C_5_: 1–0.71 < d ≤ 1 mm. Based on the data received, the cumulative PSD and median value of particle size d50 were determined. The sample of dry and ground raw material was split into nine sub-samples. Three of them were left in a dry state (moisture content 0%) and six were placed in a climatic chamber (KBF-S 115, Binder, Tuttlingen, Germany) and moisturized to 5% (3 subsamples) and to 10% (3 subsamples). Test pellets were made in a hydraulic press (P400, Sirio, Meldola, Italy) at a pressure range of 130.8–457.8 MPa. After a stabilization period (24 h), the pellets were measured (diameter and height) and weighed. Base on this, the specific density of each pellet was calculated. The mechanical durability was determined using modified standards (ISO 17831-1:2016-02) [71]. The 500 g mass of the sample, required by the standard, was met by ballast material with which the samples were tested. All measurement methods of this part of the research are described in detail in previous research work [41,43,60]. To confirm the significance of the changes in specific density and mechanical durability a statistical analysis was run. A three-way analysis (ANOVA) was conducted, and for each variant, the normality of the distribution was checked (by a Shapiro–Wilk test) with an assumption of equal variance (Brown–Forsythe test) [72,73]. For all variants, this equality was met. Finally, a one-way analysis (ANOVA) and post-hoc analysis (Scheffé’s test) were carried out, which gave an indication as to which groups showed statistically significant differences. In the final stage, a one-way analysis (ANOVA) and post-hoc analysis (Scheffé’s test) were conducted.

### 4.2. TGA Method

**Figure 18:**
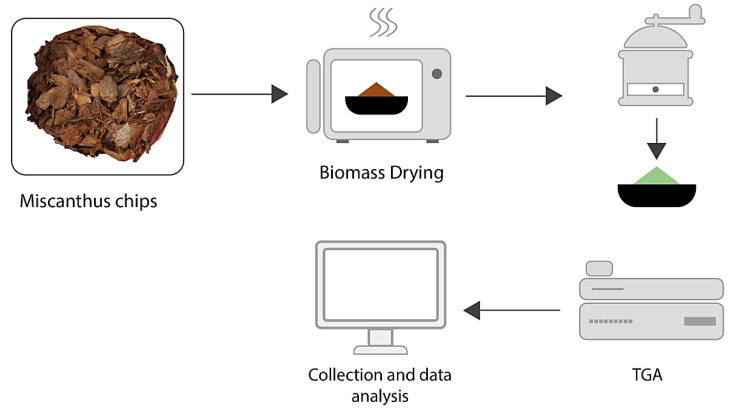
Set-up for a Miscanthus torrefaction process for the production of carbonized solid material and biocarbon as carriers for bio-fertilizers.

Before the conceptual work, a thermogravimetric analysis (TGA) was performed to find out the mass reduction and kinetics of Miscanthus in a semi-scale installation with a regimental dryer and regimental torrefaction reactor. In this research, a TG 209 Tarsus Netzsch TGA was used for the energy crop thermochemical process. A set-up with a continuous working dryer and reactor for a torrefaction process using superheated steam is planned so as to identify the proper mass loss to energy loss ratio for the highest energy density [74]. The Miscanthus carbonization process was performed using specific equipment with a thermogravimetric analyzer in an inert atmosphere, nitrogen, and argon—Figure 18.

The energy crop Miscanthus was positioned for analysis in accordance with ISO standards [75,76]. An elemental and an ultimate analysis were also carried out following carbonization. When a torrefaction process was performed, the HHVs of the Miscanthus were estimated in accordance with ISO standards [76]. The HHVs were estimated using a calorimeter bomb (Parr Instrument Co., Model 1672, Moline, IL, USA). C, H, N, S, and an O determination analysis of the Miscanthus were conducted before and after the torrefaction process, and samples were analyzed using proximate analysis. Parameters of the equipment used in experimental research are found in Table 6.

### 4.3. TGA, DSC, Kinetic Analysis, VOC, and SEM Analysis

A TGA of the Miscanthus torrefaction process was carried out using samples weighing 10 mg. Nitrogen was injected at a flow rate of 20 mL/min. To create an inert atmosphere. The Miscanthus sample was positioned in the oven of the TGA analyzer. The laboratory was configured with an electric oven and analyzer for determining the VOC and was calibrated using a precision electronic balance (with a resolution of 0.01 g) to find out the weight loss during the torrefaction process. The torrefaction temperature and residential time process conditions were registered using a computer. For thermogravimetric-spectrophotothermal TG-MS analysis, a Luxx 409 PG Netzsch thermogravimetric analyzer (Netzsch, Selb/Bavaria, Germany) was used together with an Aeolos Netzsch QMS 403D mass spectrometer (Netzsch, Selb/Bavaria, Germany). Tests were conducted under an argon atmosphere at a flow of about 25 mL/min. Since it was necessary to stabilize the weight by means of a water jacket, an initial temperature of 40 °C was determined. The mass of the biomass samples used in the biomass torrefaction process using TG-MS technique was 5.0 mg. Samples were located in Al_2_O_3_ crucibles with a diameter of 6 mm. A Differential Scanning Calorimetric analysis was performed using a DSC 3500 Sirius apparatus (Netzsch, Selb/Bavaria, Germany). The measurement methodology was as follows. A sample was prepared by weighing a high-pressure steel crucible (maximum 500 °C, maximum 100 bar) with a volume of 27 microliters without the tested biomass sample. The crucible was then weighed again with the mass of the tested material. The weighing process was based on the Radwag scale, XA 52.3 Y model working with the accuracy (52 g/0.01 mg). The high-pressure crucible was closed with a sealing press for high pressure crucibles in an oxygen atmosphere. The reference crucible was weighed empty, inserting the sample into the DSC measuring chamber. After inserting the sample and the reference crucible into the measuring chamber, the DSC 3500 Sirius with 1-414/6 software (Netzsch, Selb/Bavaria, Germany) was started and the process parameters were set. The specifications were: Initial temperature 30 °C, final temperature 500 °C; heating ratio 5, 10, and 20 K/min; gas flow; and ample weight 1.4–1.5 mg. Analysis of the results: DSC measurement data analysis was performed using Proteus version 6.1.0 (Netzsch, Selb/Bavaria, Germany). A kinetic analysis of the Miscanthus carbonization process was carried out using a thermogravimetric procedure with heating ratios of 5, 10, and 20 K/min. The Miscanthus sample masses were 10.0 ± 2 mg. A kinetic analysis was carried out in temperature ranges of 150 to 500 °C. The data were analyzed by means of Netzsch Kinetics 3 software. First, an iso-conversion analysis was conducted, which permitted a calculation of the activation energy without any knowledge of the reaction model [77]. The kinetics of the process were determined using special NETZSCH Kinetics Neo software (Netzsch, Selb/Bavaria, Germany) [78,79] by conducting a TGA analysis of the Miscanthus torrefaction process with different heating ratios of 5, 10, and 20 K/min. First, a Kissinger analysis according to ASTM E698 was performed based on the method assuming that the maximum reaction can be obtained from one step with the same degree of conversion regardless of the degree of heating. Note that this assumption is only partially correct and the resulting errors are minor. Based on Equation (1), the Kissinger method makes it possible to calculate the activation energy of the thermal decomposition reaction of a solid by plotting the logarithm of the sample heating ratio as a function of the inverse of temperature at the moment of the highest mass loss rate [80].

(1) $$f\left(T\right)=\;Ae^{-\frac{E_{e}}{RT}}$$

where *A*—pre-exponential factor (rate constant) (^1^/_s_), *E_a_*—activation energy (J/_mol_), *R*—gas constant (J/_mol·K_), and *T*—temperature (K).

### 4.4. Volatile Organic Compounds

The value of the VOC emission indicator *w_z_* in pollutants (in mg of pollutant analyzed per 1 g of combusted biofuel) was determined on the basis of:

(2) $$w_{z}=\frac{Q\cdot c_{avr}\cdot \tau }{m_{p}}$$

where: Q —air flow rate (m^3^/s), $m_{p}$—sample mass (mg), τ—sample torrefaction time (s), $c_{avr}$—average concentration of pollutants (mg/m^3^), calculated from the dependencies:

(3) $$c_{avr}=\frac{1}{\tau }\int_{0}^{t}c\left(\tau \right)dt$$

The samples were investigated by scanning electron microscopy (SEM) and energy-dispersive X-ray spectroscopy (EDX) using an SEM FEI Quanta 200FEG microscope equipped with an EDX Oxford X-Max spectrometer (Oxford Instrument, High Wycombe, UK). Measurements using the EDX technique (Oxford Instrument, High Wycombe, UK) were performed in at least 10 different spots for a given sample, and then an average atom concentration and its standard deviation were calculated for each of the identified elements. The electron energy of 20 keV was used in investigations.

### 4.5. Experimental Procedure and Device

**Figure 19:**
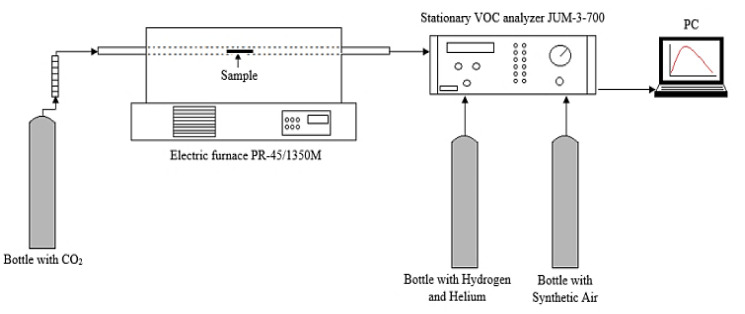
Installation for VOC analysis of “torgas” during the Miscanthus torrefaction process using an electric furnace.

**Figure 20:**
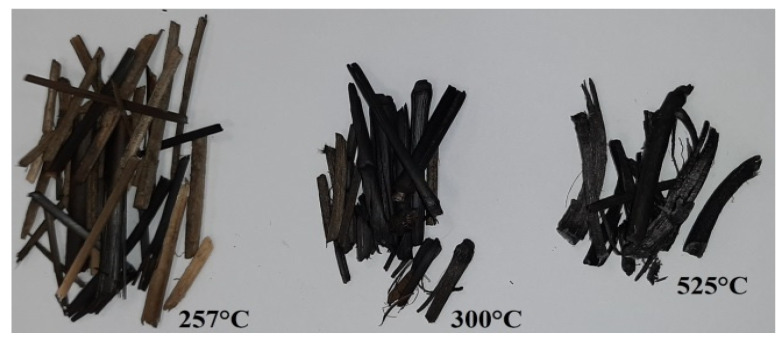
Torrefied Miscanthus samples after process at selected temperatures.

The samples were dried in the oven for 4 h at 110 °C to obtain homogeneous experimental conditions. In this research, a Miscanthus sample was processed using 2 g of substrate at atmospheric pressure. In this experiment, a horizontal tubular reactor was used with a length of 600 mm and an internal diameter of 150 mm to carry out the process of biomass carbonization, as shown in Figure 19. CO_2_ washing was carried out until the oxygen concentration in the furnace reactor fell below 1%. After flushing CO_2_ inside the reactor (2 L/min), the temperature in the furnace chamber was increased to various levels between 250 and 350 °C at a constant heating ratio of 10 K/min. When the torrefaction process attained a set temperature, the heating process was halted, and the carbon dioxide was turned off. The torrefied Miscanthus sample was immediately removed and weighed. Biomass holding time tests were carried out at different residential times starting from 0–50 min for 250, 300, 350, and 525 °C. The Miscanthus thermochemical process was carried out in an electrical oven PR-45/1350M (Przemysłowy Instytut Elektroniki, Warszawa, Poland) under a CO_2_ atmosphere. At the beginning, dried biomass with a mass of 2–2.5 g was located on a quartz glass boat, which was in turn positioned in an electrical furnace heated to the appropriate temperature for a period of one hour. The analysis was conducted at 225, 250, 275, 300, and 525 °C. The CO_2_ flow was 1/min. Simultaneously, an emission of the sum of VOC recorded in the stationary JUM FID 3-500 analyzer was performed (every 10 s). Three replicates were carried out for each temperature value. Secondly, during the Miscanthus carbonization process, the sample was weighed again to estimate the weight loss. Torrefied material can be seen in the photo in Figure 20.

### 4.6. Kinetics Models

A Friedman analysis consists of determining the logarithm of the degree of conversion and the function of temperature inverse according to Equation (4) [81]

(4) $$\ln\frac{\mathrm{dx}}{\mathrm{dt}}=\mathrm{lnA}-\frac{E_{a}}{{\mathrm{RT}}_{\max}}+\mathrm{lnf}\left(x\right)$$

The Ozawa–Flynn–Walla method is based on the integral form of the equation [81]

(5) $$G\left(x\right)=\int \frac{\mathrm{dx}}{f\left(x\right)}=\frac{A}{\beta }\overset{T}{\underset{T_{o}}{\int }}\exp\left(\frac{-x_{a}}{\mathrm{RT}}\right)\mathrm{dt}\;$$

For the Miscanthus samples analyzed, the assumption was made that the Miscanthus torrefaction process proceeds in one step according to the scheme:

A−1→B

where B—is the torrefied Miscanthus.

In linear form, the thermokinetic equation takes the form [81].

(6) $$\ln\frac{g\left(X\right)}{T}=\ln\frac{A}{\beta }-\frac{E}{\mathrm{RT}}$$

## 5. Conclusions

This article investigates the conditions of the Miscanthus drying and torrefaction processes used to obtain two types of products. In this work, we primarily examined the influence of the material drying temperature, moisture content, and compaction pressure on test pellets’ DE and DU. Furthermore, this varies depending on the material tested. The drying temperature has no effect on the course of DE changes but has a slight effect on DU changes (drying at 140 °C allows DU 97.5% at 240MPa and 10% moisture content to be obtained). DE and DU depend on moisture content and compression pressure. Higher pressure levels do not cause significant differences. It has been shown that moisture content has a major influence on the course of DE and DU changes. Although the drying temperature changes the properties of the material, these changes do not significantly affect the compaction process (there is no clear dependence of DE and DU on the drying temperature). This is a result which means that the material dried over a wide temperature range can be used for the compaction stage without the risk of losing the quality parameters of the granulates. This allows the right drying temperature to be selected for the material preparation processes, thus reducing the effort required for these processes. For example, the drying temperature significantly influences the grinding process. Jewiarz and others have shown that regardless of the tested material (Scots pine, European beech, Giant Miscanthus, and Cup plant) Sokolowski’s grinding criterion G0.25 shows that the energy inputs in the grinding process decrease as the raw material drying temperature increased. Secondly, the authors quantified various parameters of the biomass carbonization process like it was done on different biomass in [82,83,84]. These included kinetics, optimal temperature of the biomass roasting process, and residence time for specific mass loss ratios [85,86,87,88,89,90,91,92]. Additionally, high VOC concentrations in the tracks show that torgas can be used as a heat source for the roasting process. It was found that (257 °C, 9 min under this isothermal conditions) temperature at which during Miscanthus torrefaction process we are in the closest temperature at which mass loss of torrefied material have lost 30% of it original mass which according to the previous studies on many different biomass torrefaction process the most optimal mass loss to have the highest increase in caloric value (with a 30% mass loss we are losing only approximately 10% of energy of the torrefied biomass) [93,94,95,96,97]. At (300 °C, 6 min) there was observed closest to 50% mass loss which according to the literature is one of the most optimal mass loss for the biochar production as additive/carrier of C for fertilizers. At (525 °C, 5 min) the mass loss was closest to 75% mass loss which from the process and it economic point of view is reasonable temperature for the production of biosorbents/activated carbons [98,99]. It can be also added that this can impact the bio economy by the fact that an optimal mass loss ratio to energy loss for different energy crops during the torrefaction process has a significant impact on both carbonized solid biofuel and biochar as carriers for fertilizer prices (too much or too little heat for the process has a significant impact on the final product prices) [100]. In the next stage of the future research on the Miscanthus torrefaction process, there will be a focus on building an installation for a continuous torrefaction process using superheated steam and using kinetics data from this article to design a biomass rotary dryer run on hot air and counter-flow reactor for torrefaction process run on dry steam (superheated steam). In the spring of 2021, a new set-up will be built and experimental research will be carried out on the conditions of the torrefaction process of Miscanthus and other woody and agri-biomass for the production of carbonized solid biofuels, biocarbon as a carrier for organic fertilizers, and activated carbon.

## Figures and Tables

**Table 1:** Three-way ANOVA results for specific density.

	SS	df	MS	F Value	*p*-Value
Intercept	216,951,606	1	216,951,606	2,465,092	0.00
Temperature	65	2	33	0	0.69
Moisture	589,762	2	294,881	3351	0.00
Pressure	666,172	5	133,234	1514	0.00
Temperature x Moisture	4045	4	1011	11	0.00
Temperature x Pressure	1682	10	168	2	0.046
Moisture x Pressure	91,084	10	9108	103	0.00
Temperature x Moisture x Pressure	4169	20	208	2	0.0016
Error	14,258	162	88		

**Table 2:** Three-way analysis of variance ANOVA—DU.

	SS	df	MS	F Value	*p*-Value
Intercept	1,523,952	1	1,523,952	7,800,727	0.00
Temperature	687	2	343	1758	0.00
Moisture	29,810	2	14,905	76,294	0.00
Pressure	47,372	5	9474	48,497	0.00
Temperature x Moisture	136	4	34	174	0.00
Temperature x Pressure	211	10	21	108	0.00
Moisture x Pressure	29,103	10	2910	14,897	0.00
Temperature x Moisture x Pressure	233	20	12	60	0.00
Error	32	162	0		

**Table 3:** Pre-exponential coefficient and activation energy as a function of the degree of conversion by the Ozawa–Flynn–Walla method for Miscanthus.

Fract. Mass Loss	Activation Energy (kJ/mol)	Log (A/s^−1^)
0.02	115.33 ± 34.46	8.05
0.05	105.80 ± 23.54	6.87
0.10	109.45 ± 18.57	7.27
0.20	99.07 ± 18.21	6.27
0.30	100.06 ± 21.66	6.30
0.40	104.41 ± 25.94	6.63
0.50	119.38 ± 28.20	7.93
0.60	145.97 ± 27.97	10.23
0.70	186.50 ± 22.91	13.67

**Table 4:** Proximate and ultimate analysis of Miscanthus before and after the torrefaction process.

Energy Crop	M_ad_ (%)	C_d_ (%)	N_d_ (%)	H_d_ (%)	S_d_ (%)	O_d_ (%)	Cl_d_ (%)	V_d_ (%)	A_d_ (%)	HHV_d_ (MJ/kg)	Mass Loss (%)
Miscanthus	5.3	48.5	0.27	6.20	0.05	42.56	0.115	91.29	2.3	15.82	-
Torrefied Miscanthus:											
(257 °C, 9 min)	2.8	54.37	0.19	5.37	0.05	36.06	0.014	73.37	3.94	21.70	30.69
(300 °C, 6 min)	1.5	57.04	0.16	4.93	0.05	32.36	0.013	61.11	5.44	26.72	43.56
(525 °C, 5 min)	1.1	59.29	0.14	3.69	0.04	27.61	0.012	44.27	9.21	27.69	76.24

M—moisture content, C—carbon content, N—nitrogen content, H—hydrogen content, S—sulfur content, O—oxygen content, Cl—chlorine content, V—volatile matter content, A—ash content, HHV—higher heating value, _ad_—air dried state, and _d_—dry state.

**Table 5:** Content of mineral elements in the torrefied Miscanthus and untreated Miscanthus at three different temperatures.

	Mineral Composition of Torrefied Miscanthus (257.5 °C)		Mineral Composition of Torrefied Miscanthus (300 °C)		Mineral Composition of Torrefied Miscanthus (525 °C)	
Element	Arithmetic Average	Standard Deviation	Aritmetic Average	Standard Deviation	Arithmetic Average	Standard Deviation
C	68.33	1.175	73.98	1.742	83.75	2.195
O	29.78	1.535	24.11	2.552	14.23	2.556
Al	0.09	0.017	0.87	0.749	0.07	0.016
Si	1.49	0.443	1.05	0.260	0.54	2.508
K	0.08	0.064	0.08	0.015	0.82	0.356
Ca	0.07	0.017	0.19	0.038	0.13	0.040
Mg	0.06	0.10	0.07	0.000	0.11	0.028
S	0.06	0.000	0.23	0.052	0.15	0.008
P	0.11	0.033	0.12	0.040	0.17	0.049

**Table 6:** Equipment used in experimental research on the Miscanthus torrefaction process.

Device	Model	Producer	Device Parametr
**Thermogravimeter**	TG 209 F3 Tarsus	Netzsch (Germany)	Heating ratio: 0.001 K/min to 200 K/min TGA resolution: 0.1 µg
**Differential Scanning Calorimeter DSC Analyzer**	DSC 3500 Sirius	Netzsch (Germany)	Heat Flux sensor, Temperature range: −170 °C to 600 °C Heating ratios: 0.01 K/min to 100 K/min Temperature accuracy: 0.1 K Enthalpy accuracy: Generally < 2%
**Laboratory electrical tube furnace**	PR-45/1350M	Przemysłowy Instytut Elektroniki PIE (Poland)	Maximum temperature of measurement: 1350 °C Accuracy of temperature control: +/− 5 °C Furnace heating speed: 100 °C/8 min
**Stationary analyzer VOC**	FID 3-500	J.U.M. Engineering (Germany)	Measurement method: Continuous flame ionization detection (FID) Measurement range: 0–100 000 ppm Tenderness: Maximum 1 ppm CH_4_ for full scale Response time: 1.2 s Linearity: 1%
**Calorimeter**	PARR 6400	Parr Instrument Company (USA)	Precision class instrument: 0.1% Temperature Resolution: 0.0001 °C Calorie sample range: 5000–8000 Linearity across operating range: 0.05%
**Energy Dispersive X-ray Spectroscopy**	EDX Oxford X-Max spectrometer-SEM FEI Quanta 200FEG microscope	Oxford Instruments (USA)	EDX elemental map of a gallium arsenide single crystal sample with <011> orientation, obtained using HD-2700-spherical aberration corrector, dual SDDs. The acceleration voltage is 200 kV, the pixel number is 128 by 96, the acquisition time is 12 min.

**Figure A1:**
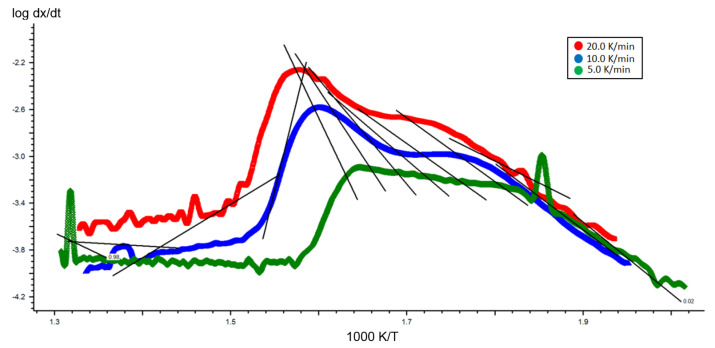
Log dx/dt graph as a function of temperature reverse using the Friedman method for Miscanthus.

**Figure A2:**
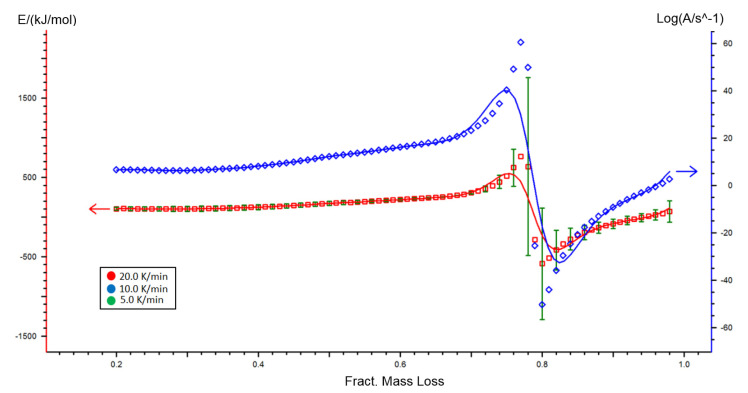
Activation energy and pre-exponential coefficient as a function of the degree of conversion by the Friedman method for Miscanthus.

**Figure A3:**
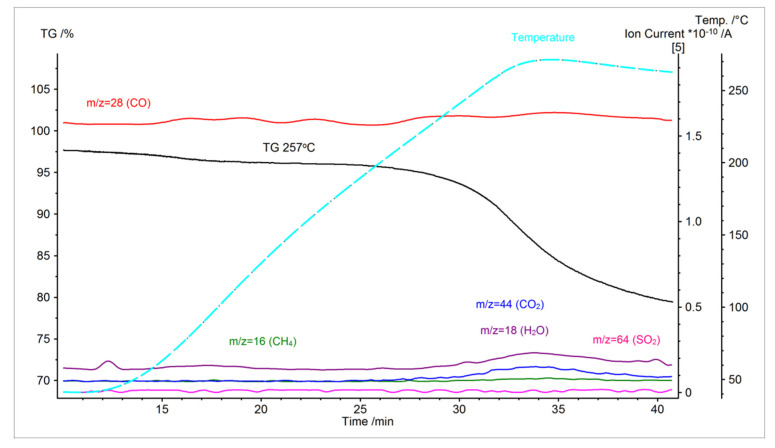
Thermogravimetric and ion current curves for the Miscanthus torrefaction process up to 257.5 °C.

**Figure A4:**
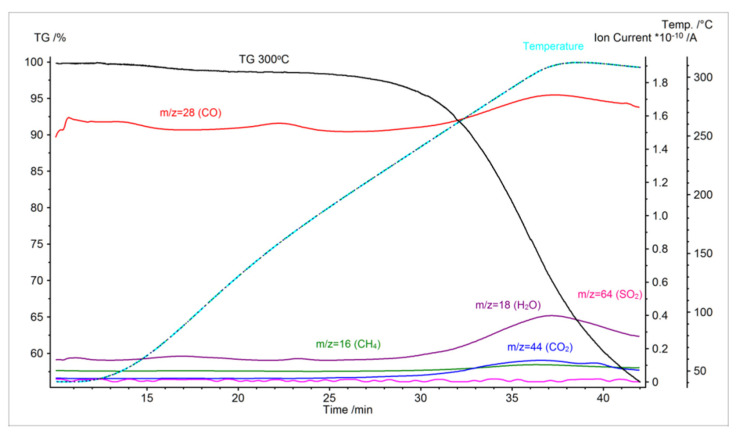
Thermogravimetric and ion current curves for the Miscanthus torrefaction process up to 300 °C.

## Data Availability

The data presented in this study are available on request from the corresponding author.

## Sample Availability

Samples of the compounds of dry and torrefied Miscanthus are available from the authors.

## Appendix A

The activation energy value obtained for Miscanthus is 27 kJ/mol. A lower error in this method was 13 kJ/mol. The level of conversion and speed of reaction were both derived from the formulas. Due to the fact that f(x) is constant for a given conversion, the log with dx/dt as a function of temperature inverse gives straight lines with a slope equal to E_a_/R. Figure A1 shows logs of the dx/dt logarithm as a function of temperature inverse determined by Friedman’s method for Miscanthus.

Using the Friedman method, it is possible to show the value of pre-exponential coefficient and activation energy as a function of the level of conversion. Figure A2 shows these graphs for a sample of Miscanthus.

Analysis of Figure A2 gives the activation energies of Miscanthus which differ depending on the extent of conversion. In Table A1 (Appendix A), we can find summarized the theoretical equations of the solid-state distribution models of g(x), which we have taken to determine the best fit.

**Table A1:** Pre-exponential coefficient and activation energy as a function of the degree of conversion by the Friedman method for Miscanthus.

Fract. Mass Loss	Activation Energy (kJ/mol)	Log (A/s^−1^)
0.02	108.12 ± 26.14	7.17
0.05	103.12 ± 18.65	6.65
0.10	91.07 ± 20.76	5.55
0.20	101.12 ± 20.73	6.40
0.30	102.77 ± 32.18	6.39
0.40	126.43 ± 31.52	8.42
0.50	170.58 ± 23.37	12.21
0.60	216.56 ± 12.43	16.07
0.70	301.28 ± 16.43	23.02

The activation energy and pre-exponential coefficient as a function of the level of conversion by the Ozawa–Flynn–Walla method for Miscanthus can be found in Table 3. On Figure A3 and Figure A4 thermogravimetric and ion current curves for the Miscanthus torrefaction process up to 257.5 °C and up to 300 °C are presented.
